# Artificial Fecundation

**Published:** 1871-03

**Authors:** 


Artificial Fecundation.—Dr. Girault, of Paris, has made 27 attempts at impregnating women in whom conception in the natural way was impossible, by injecting into the uterus, at the proper time, some of the husband's semen. Ten of the cases resulted in conception, and others would, he thinks, had the parties persisted in their efforts and allowed him to continue his endeavors.

A uterine catheter is the only instrument he uses. He places the sperm within this, inserts its point into the uterine canal, and blows the fluid into the womb cavity.—*Medical and Surgical Reporter.*

